# Cooperation between arbuscular mycorrhizal fungi and plant growth-promoting bacteria and their effects on plant growth and soil quality

**DOI:** 10.7717/peerj.13080

**Published:** 2022-03-21

**Authors:** Lu Yu, Hui Zhang, Wantong Zhang, Kesi Liu, Miao Liu, Xinqing Shao

**Affiliations:** 1 College of Grassland Science and Technology, China Agricultural University, Beijing, China; 2 Qinghai Provincial Key Laboratory of Adaptive Management on Alpine Grassland, Qinghai, China

**Keywords:** Arbuscular mycorrhizal fungi (AMF), Plant growth-promoting rhizobacteria (PGPR), Root morphology, Soil physicochemical properties, Plant-microbe interaction

## Abstract

The roles of arbuscular mycorrhizal fungi (AMF) and plant growth-promoting rhizobacteria (PGPR) in improving nutrition uptake and soil quality have been well documented. However, few studies have explored their effects on root morphology and soil properties. In this study, we inoculated *Elymus nutans* Griseb with AMF and/or PGPR in order to explore their effects on plant growth, soil physicochemical properties, and soil enzyme activities. The results showed that AMF and/or PGPR inoculation significantly enhanced aboveground and belowground vegetation biomass. Both single and dual inoculations were beneficial for plant root length, surface area, root branches, stem diameter, height, and the ratio of shoot to root, but decreased root volume and root average diameter. Soil total nitrogen, alkaline phosphatase, and urease activities showed significant growth, and soil electrical conductivity and pH significantly declined under the inoculation treatments. Specific root length showed a negative correlation with belowground biomass, but a positive correlation with root length and root branches. These results indicated that AMF and PGPR had synergetic effects on root morphology, soil nutrient availability, and plant growth.

## Introduction

Almost 25% of the Earth’s biodiversity is composed of soil microorganisms that have interacted with animals, plants, and soil in ecosystems around the world for millions of years (Fierer, 2017; Wagg et al., 2019; Whitman, Coleman & Wiebe, 1998). Numerous beneficial microorganisms play critical roles in biogeochemical circulation that fulfill global carbon (C) and nutrient cycling that allow ecosystems to function and improve their productivity. However, the plant-microbe interaction has still been undervalued in studies on the direct plant-soil feedback effects and links between plant communities and soil microbes, such as in nutrient acquisition and hormone stimulation (Fierer, 2017; Morgan, Bending & White, 2005). Plant diversity has been shaped by microorganisms *via* their symbionts in terrestrial ecosystems (Wagg et al., 2014). Generally, rhizosphere microbes can assist the growth and development of plants by recycling nutrients, producing hormones, improving tolerance toward potentially hazardous compounds, keeping the soil healthy, and exercising other indispensable functions such as soil formation and decomposition of organic matter (Wallenstein, 2006). When plants lack essential mineral elements such as phosphorus (P) or nitrogen (N), this kind of symbiont can enhance and benefit plant growth. Soil microorganisms, including mutualists and pathogens, drive abiotic properties and regulate individual plant growth and species coexistence with successive mutual interactions (Li et al., 2020). This plant-associated microbiota involves various groups of organisms, including bacteria, archaea, and fungi, acting as a symbiont or pathogen (Berendsen, Pieterse & Bakker, 2012; Hussain & Khan, 2020; Vorholt, 2012).

Arbuscular mycorrhizal fungi (AMF), assigned to the Glomeromycotina phylum, are one of the most important components of the soil ecosystem. They maintain symbiotic relationships with over 70% of terrestrial plants and provide nutrients and water to plants in exchange for sugars through their arbuscules, which is where the exchange of necessary nutrients between host plants and fungi occurs (Schussler, Schwarzott & Walker, 2001*; Wagg et al., 2019*). AMF have the capacity to expand the exhaustion zone using an extensive hyphal network to acquire extra water and nutrients that can significantly improve a host plants’ fitness (Bonfante & Genre, 2010; Ezawa & Saito, 2018; Field & Pressel, 2018; van der Heijden et al., 2015). AMF also enhance the ability of host plants and adjacent plants that are connected with common mycorrhizal networks (CMN) to resist drought, heavy metals, and pathogens (Bonfante & Genre, 2010; Giovannetti et al., 2001; Pepe, Giovannetti & Sbrana, 2016). The area around the mycorrhizal hyphae, called the hyphosphere (Patel, Thakkar & Subramanian, 2015; Rasmann et al., 2017), contains helper bacteria that promote the plant-mycorrhizal fungus symbiotic associations, plant-growth-promoting rhizobacteria (PGPR) that work collaboratively with AMF to facilitate plant growth and productivity, and mycophagous bacteria that are dependent on fresh hyphaes (Yuan et al., 2021). PGPR include *Azospirillum*, *Pseudomonas*, *Azotobacter*, *Klebsiella*, *Enterobacter*, *Alcaligens*, *Arthrobacter*, *Burkholderia*, and *Bacillus* spp., which are important members of the plant-associated microbiome (Bashan et al., 2014; Muzammil et al., 2014). *Bacillus spp*. promotes plant growth by fixing N, solubilizing and mineralizing P and other nutrients, stimulating phytohormones, producing siderophores, inducing systemic resistance (ISR), and enhancing their tolerance to abiotic stresses (Bonfante & Genre, 2010; Saxena et al., 2020). Bacteria rely on lignin and cellulose-hydrolysed fungus to produce primary bacterial materials and provide nourishment to fungus for exchange (Clausen, 1996; Romaní et al., 2006). Fungal hyphae form hyphal networks to connect soil patches and build “fungal hyphae highways” for bacteria to transfer substrates (Warmink et al., 2011).

Numerous studies have shown that utilizing PGPR and AMF is a feasible ecological approach to enhance soil health and plant productivity (Aini, Yamika & Ulum, 2019; Vessey, 2003). AMF and PGPR symbionts can enhance resistance to salinity by shifting individual root morphology and root-to-shoot communication, keeping ion homeostasis, diminishing oxidative damage, and increasing photosynthetic capacity (Chandrasekaran et al., 2014; Egamberdieva et al., 2017; Pan et al., 2019), as well as significantly elevating aboveground biomass, stem branches, and plant height (Pan et al., 2020). Additionally, the combination of AMF and PGPR promoted the projected area, total volume, and total root length of trifoliate orange under limited P conditions (Wang et al., 2016), and aboveground organs under deficient organic N (Saia et al., 2015), N, P, potassium (K), and sulfur (S) concentrations in the rhizospheres of onion and maize (Mohamed et al., 2014).

However, there is still a lack of detailed insight and evidence to verify the key functions of AMF, PGPR, and their combined effects on the growth and development of dominant species of the grassland community. *Elymus nutans* Griseb is a dominant perennial species in the Qinghai-Tibet Plateau, China (Chu et al., 2016) that plays an important role in animal husbandry and the ecological conservation of this region. Therefore, this study was conducted to investigate the effects of AMF and PGPR on plant traits of *Elymus nutans* Griseb and the surrounding soil properties.

We hypothesized that: (1) AMF and PGPR could mutually symbiose and enhance the plant growth of *Elymus nutans* Griseb, and (2) the co-existence of AMF and PGPR could improve plant traits and soil quality better than their individual applications.

## Materials and Methods

### Plant materials

This pot experiment had a fully randomized one-factor experimental design with four treatments. The soil was excavated (0–15 cm depth) in March 2020 from the Qinghai-Tibet Plateau, China, (53°19′2.44″N and 13°51′48.03″E), which has a typical plateau continental climate and clay loam type soil. Precipitation is greatest between June and September (Wei et al., 2021). The sampled soil was air-dried for about two weeks and sieved through a 2 mm screen to ensure homogeneity. The sieved soil was sterilized at 121 °C for 2 h and put into a 2 L pot (17 cm height × 12 cm internal diameter × 14 cm external diameter) for the later experiment.

*Elymus nutans* Griseb seeds were collected from Haibei Autonomous Prefecture, Qinghai Province, China (36°55′N, 100°57′E, 3,029 m a.s.l.). Before sowing, the seeds were surface-sterilized with 10% H_2_O_2_ and rinsed three times with sterile water (Schweiger, Baier & Mueller, 2014). The seeds were then sowed in a 3:2 mixture of clay:sand and laid in the climate chamber (20 °C, 12/12 h: light/dark, 60–70% r.h.). After two weeks, 50 *Elymus nutans* Griseb seedlings were transferred into experimental pots. They were stored in the dark at 4 °C for 3 d and then transferred to a greenhouse (Tomczak, Schweiger & Müller, 2016) where they were watered twice a week with a standard quantity of water (1,800 ml/week were added in each pot based on the experimental pot size).

### AMF inoculum

The inoculum of mycorrhizal fungus *Funneliformis mosseae* (accession no. BGCYN05), obtained from the Beijing Academy of Agriculture and Forestry Sciences, was isolated from red clover and contained spores, mycelium, sand, and root fragments The inoculant used in this experiment was a rhizosphere soil mixture of spores, extrarhizoma hyphae, and root segments of infected plants, and contained 129 spores per gram. The mycorrhizal fungus spores were placed almost 2 cm below the soil surface before sowing the seeds.

### PGPR inoculum

The *Bacillus megaterium* (accession no. ACCC10011) that were provided by the Institute of Agricultural Resources and Regional Planning (ACCC). *Bacillus* was grown in 10 g of beef extract peptone AGAR medium with peptone, 3 g of beef extract, 5.0 g of NaCl, and 1,000 mL of distilled water, then 20 g of agar was added. We sterilized the AGAR medium at 121 °C for 30 min, and *Bacillus* was grown overnight with constant shaking at 220 rpm (Constantino et al., 2008). We regulated the cell suspension to 10^9^ CFU mL^−1^ and then used it as a standard inoculum. The seeds were immersed in a bacterial suspension before sowing, and the seedling were inoculated with 20 ml of same bacterial suspension after sowing.

### Experimental design

Four treatments were used to explore the effects of fungus-bacteria symbiont on *Elymus nutans* Griseb, including one dual inoculation treatment (30 g-AMF inoculum and 25 ml-*Bacillus* suspension), two single inoculation treatments (25 ml-*Bacillus* suspension and 30 g AMF inoculum, respectively), and one controlled treatment (AMF and PGPR were both autoclaved). Each treatment included six replicates.

### Determination of parameters

#### AMF colonization

Mycorrhizal infection was examined using Kormanik & McGraw’s (1982) method. The receiver plant roots were randomly collected per treatment, washed with distilled water, cut into 1 cm segments, immersed in 10% KOH, and heated in water under 90 °C for 60 min. The roots segments were watered to eliminate the alkali, and the remaining alkali was neutralized in 2% hydrochloric acid for 10 min. Then we added 0.01% acid fuchsin to stain the root segments, heated them in 90 °C for 30 mins after separating them from the acid solution, and then immersed them in the mixture solution of glycerol/lactic/water acid (1:1:1) for 24 h to destain them. After that, the treated samples were observed using a regular optical microscope in 40× to qualify the levels of mycorrhizal colonization (Dalpé & Séguin, 2013).

$${\rm{Mycorrhizal}}\;{\rm{colonization}}\;(\% ){\rm{ = number}}\;{\rm{of}}\;{\rm{infected}}\;{\rm{root}}\;{\rm{bits/total}}\;{\rm{number}}\;{\rm{of}}\;{\rm{root}}\;{\rm{bits}}\;{\rm{observed}} \times {\rm{100}}\% $$

### Analysis of parameters

#### Plant parts

The plant samples were separated into shoots and washed roots, dried at 60 °C for 72 h, and weighed to determine the biomass of dry shoots and roots (Bourles et al., 2020). To determine the total C and N content, leaf tissues were milled with a ball mill (Retsch MM400; Retsch, Haan, Germany), 0.15 g of the sieved plant sample was weighed, wrapped in the tin cup required by the instrument, and measured using an elemental analyzer (Vario MAX CNS; Elementar, Hanau, Germany). The plants were thoroughly washed with deionized water to remove all soil particles and dust, and were then divided into two parts: shoot and root parts. The shoot and root parts were then scanned using EPSON Perfection V700 PHOTO and WinRHIZO Pro software (Regent Instruments Inc., Quebec, Canada) to look at the shoot size, root/shoot ratio, root surface area, root length (mm), mean root diameter (mm), and root branches.

#### Soil parts

Soil total P content was measured using the sodium hydroxide melting-molybdenum antimony colorimetric method (Yang et al., 2018). Soil total C and N were milled using a ball mill (Retsch MM400; Retsch, Haan, Germany), and weighing 0.15 g of the sieved soil sample, wrapping it in the tin cup required by the instrument, and using the elemental analyzer (Vario MAX CNS, Elementar, Hanau, Germany). Soil ammonium N (NH^4+^-N) and nitrate N (NO^3−^-N) levels were measured after extraction using 50 mL of 2 mol/L KCl on a 10 g subsample and a potassium chloride leaching-flow-solution analyzer. Soil available P was measured using Xiao et al. (2018) and Yang et al. (2018). Soil urease enzyme (UE), and alkaline phosphatase activities (ALP) were examined using the Solarbio soil urease kit (Solarbio, BC0120, Beijing, China) and soil alkaline phosphatase kit (Solarbio, BC0280, Beijing, China), respectively.

### Data analysis

Plant traits, soil properties, and soil enzyme activities were analyzed using a one-way analysis of variance (ANOVA) test in the “agricolae” package of R software. The correlation between specific root length and plant traits was analyzed using the “corrplot” package. The statistical analysis figures were produced with R software version 4.1.0 (Frew, Powell & Johnson, 2020). The differences were considered to be significant at a 0.05 level.

## Results

### Plant traits and nutrient uptake

**Figure 1:**
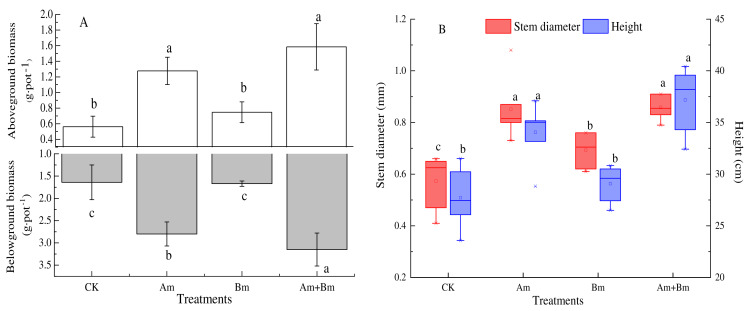
Effect of different inoculations on the aboveground and belowground biomass (A) and stem diameter and height (B). Different letters represent significant differences at *P* = 0.05 using one-way ANOVA. The error bars in the figure legends represent the standard deviation. CK, no inoculation; (Am), only AMF inoculation; Bm, only Bacillus inoculation; Am + Bm, both symbionts.

There were significant differences across all treatments (*P* < 0.05). The aboveground and belowground biomass from dual inoculation with AMF and *Bacillus* had maximum values of 3.15 g·pot^−1^ and 1.59 g·pot^−1^, respectively. Compared with the control group, mixed inoculations were twice as high for aboveground biomass and four times higher for belowground biomass (Fig. 1A).

Stem diameter and plant height with inoculations were greater than those with no inoculation (CK) (*P* < 0.05) (Fig. 1B), and both stem diameter and height were significantly highest in the dual inoculation (Am + Bm) (*P* < 0.05) group, followed by only AMF inoculation (Am), and least in only *Bacillus* inoculation (Bm). The stem diameter and height values ranged from 0.57 mm to 0.86 mm and 27.72 to 37.19 cm, respectively. Single and dual inoculations (Table 1) increased the concentration of plant total C and N, but decreased plant total P compared with the CK group. There were no significant differences in plant total C and N (*P* > 0.05).

**Table 1:** Effect of different inoculations on plant traits and soil properties.

Treatments	Plant traits			Soil properties			
	Total carbon (%)	Total nitrogen (%)	Total phosphorus (g·kg^−1^)	Total carbon (%)	Total nitrogen (%)	Total phosphorus (g·kg^−1^)	Available phosphorus (g·kg^−1^)
CK	42.64 ± 0.33a	2.10 ± 0.16a	0.06 ± 0.02b	3.43 ± 0.12a	0.21 ± 0.02ab	0.43 ± 0.14a	2.26 ± 0.18a
Am	42.96 ± 0.41a	1.98 ± 0.17a	0.02 ± 0.002a	3.45 ± 0.06a	0.22 ± 0.02a	0.46 ± 0.06a	2.21 ± 0.13a
Bm	42.65 ± 0.38a	1.97 ± 0.17a	0.04 ± 0.01b	3.43 ± 0.06a	0.19 ± 0.01b	0.48 ± 0.04a	2.12 ± 0.10a
Am + Bm	42.64 ± 0.64a	1.89 ± 0.22a	0.03 ± 0.003a	3.46 ± 0.04a	0.21 ± 0.01ab	0.47 ± 0.01a	2.85 ± 0.46a

**Note:**

Different letters represent significant differences at *P* = 0.05 in using one-way ANOVA. Data (average ± SE, *n* = 6) in the same column with different letters indicates significant differences according to LSD test (*P* < 0.05). CK, no inoculation; (Am), only AM fungi inoculation; Bm, only bacillus inoculation; Am + Bm, both symbionts.

Dual inoculation with AMF and PGPR did not significantly affect mycorrhizal colonization (Table 2). There were statistically significant differences across the four treatments in root length, root surface area, root branches, and root average diameter (*P* < 0.05). Inoculations with AMF and/or PGPR enhanced root length, root surface area, shoot to root ratio, and root branches (*P* < 0.05). However, root volume and root average diameter showed the greatest discrepancy in the Am and Bm groups, respectively. In the presence of AMF, the root length, root surface area, and root branches were much greater than in the other groups, and Bm had a positive effect on root surface area and root to shoot ratio (*P* < 0.05).

**Table 2:** Effect of different inoculations on the root length and root surf area (a) and root/shoot radio and root branches (b) and root volume and root average diameter.

Treatments	Root colonization	Root length cm	Root surf area cm^2^	Root volume cm^3^	Root branches	Root ADV mm	Root to shoot ratio	Specific root length
CK	0 ± 0b	318.56 ± 68.5c	786.34 ± 82.28b	161.18 ± 14.03a	1,313.11 ± 334.57b	8.41 ± 1.13a	0.35 ± 0.07b	5.94 ± 1.88a
Am	59.77 ± 10.44a	610.87 ± 77.2a	883.65 ± 64.79a	105.27 ± 14.55c	4,160.78 ± 1594.83a	4.75 ± 0.55c	0.45 ± 0.04a	4.82 ± 0.58a
Bm	0 ± 0b	446.82 ± 100b	880.37 ± 78.69a	146.39 ± 34.53b	2,265.39 ± 879.79 b	6.64 ± 1.43b	0.44 ± 0.06ab	6.01 ± 1.14a
Am + Bm	58.07 ± 9.37a	712.70 ± 113a	859.8 ± 51.54ab	86.32 ± 17.16c	3,629.00 ± 667.67a	4.00 ± 0.67c	0.51 ± 0.12a	4.67 ± 1.31a

**Note:**

Different letters represent significant differences at *P* = 0.05 in using one-way ANOVA. CK, no inoculation; Am, only AM fungi inoculation; Bm, only bacillus inoculation; AM + BM, both symbionts.

### Soil properties

**Figure 2:**
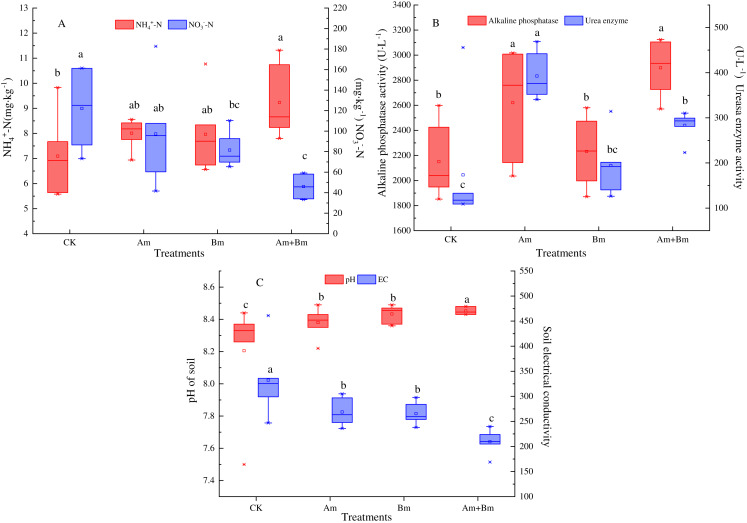
Effect of different inoculations on the soil ammonium nitrogen and nitrate nitrogen content (A), alkaline phosphatase and urea enzyme (B), and pH and electrical conductivity (C). Different letters represent significant differences at *P* = 0.05 using one-way ANOVA. The error bars in the figure legends represent the standard deviation. CK, no inoculation; (Am), only AMF inoculation; Bm, only Bacillus inoculation; Am + Bm, both symbionts.

Single AMF inoculation significantly improved the content of soil total N (Table 1), which was decreased in the Bm group (*P* < 0.05). Am and Bm treatments showed less soil available P compared with the control group. Inoculations significantly enhanced the content of soil NH^4+^-N but decreased soil NO^3−^-N (Fig. 2A).

The ALP and UE activity results showed that single or multiple inoculations enhanced ALP activities, and ALP was greater in the presence of AMF than in CK and Bm treatments, which increased to 2,620.76 U·L^−1^ and 2,899.35 U·L^−1^, respectively. In the meantime, AMF enhanced UE activities more than other treatments by about 218.07 U·L^−1^ (*P* < 0.05) (Fig. 2B). The dual inoculation group (Am + Bm) had the lowest pH value but the highest electrical conductivity, while the control group had the opposite results (Fig. 2C).

**Figure 3:**
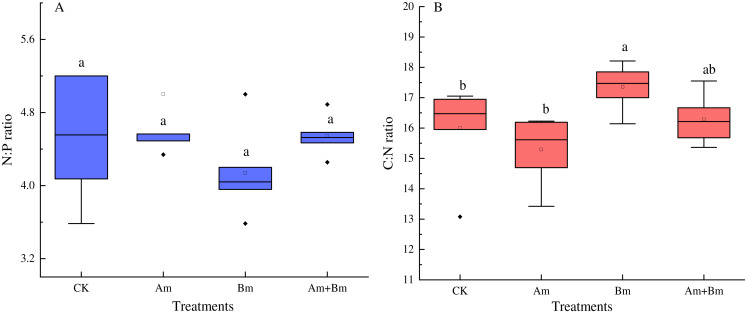
Effect of different inoculations on the N:P ratio (A) and C:N ratio (B). Different letters represent significant differences at *P* = 0.05 using one-way ANOVA. The error bars in the figure legends represent the standard deviation. CK, no inoculation; (Am), only AMF inoculation; Bm, only Bacillus inoculation; Am + Bm, both symbionts.

The N:P ratio was not significantly influenced by any inoculations (Fig. 3A), but there were significant differences in C: N ratio across all treatments (*P* < 0.05) (Fig. 3B), and PGPR inoculation promoted the C:N ratio, but decreased N:P ratio.

### Correlations among traits and related variables

**Figure 4:**
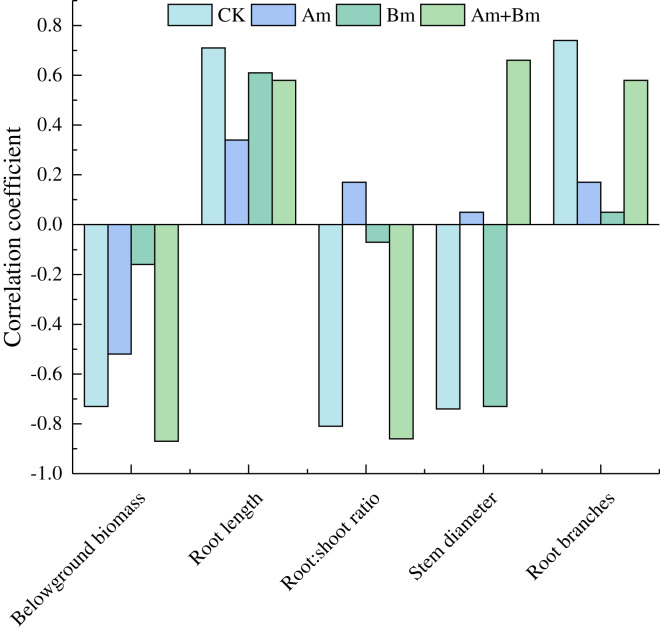
Pearson correlations in specific root length and root variables across different inoculations. CK, no inoculation; (Am), only AMF inoculation; Bm, only Bacillus inoculation; Am + Bm, both symbionts.

Pearson analysis between specific root length and plant traits showed that specific root length was positively correlated with root length and root branches, but was negatively correlated with belowground biomass (Fig. 4).

## Discussion

### Plant trait responses to inoculations

Plant-soil microorganism interactions play an essential role in nutrient acquisition and ecosystem functions. However, few studies have focused on soil microorganism responses, especially fungi-bacteria co-occurrence, to plant root growth. In this study, we quantified how plant-associated microbial symbionts affected plant growth and traits, as well as changes in soil physicochemical properties. The aboveground and belowground biomass of the experiment species were significantly promoted in the presence of AMF and *Bacillus* compared to a single symbiont (Fig. 1). The AMF-PGPR symbiont increased P and N acquisition (Aliasgharzad, Neyshabouri & Salimi, 2006), indicating the positive and synergistic effects of combining AMF and PGPR in the host plant (Bourles et al., 2020; Shockley, McGraw & Garrett, 2004; Xie et al., 2020). PGPR + AMF also influenced AMF colonization, which was suggested by earlier studies that found that multiple inoculations increase AMF colonization (Aini, Yamika & Ulum, 2019; Constantino et al., 2008; Juge et al., 2012) as well as root length and resource acquisition. In general, higher root hydraulic conductivity and proliferation rate were demonstrated by a higher specific root length (Rewald Email et al., 2013). We observed that specific root length decreased with microbial inoculations (but was increased by Bm). This may be due to that AMF mycelium replace the absorption function of the root system, and that the specific compounds secreted by PGPR and AMF provide more nutrition and eliminate toxic ions in the rhizosphere, which facilitates the host to develop a thicker root system and larger absorption area (Brundrett, 2002; Solomon & Rajeshkanna, 2013). Therefore, it is not necessary for *Elymus nutans* Griseb inoculated with AMF and PGPR to develop a larger specific root length as a substitute for its diameter in order to obtain more resources (Hodge, 2004).

Our results found that height, root, biomass, and root surface area increased following the mixed inoculation, which confirmed the results found by Toro, Azcon & Barea (1997), Ma, Rajkumar & Freitas (2009) and Xie et al. (2020). Additionally, our findings also showed that the presence of AMF and PGPR promoted biomass and N and P accumulation in plant tissues. This is possibly due to AMF and PGPR effectively influencing the calculation of N compounds (like amino acids and soluble proteins) in the host plant (Xie et al., 2020), and their coexistence mediates the production of phytohormones or enzymatic activities to further root evolution and growth (Abdel-Rahman & El-Naggar, 2014), as well as enhance the foundation and development of rhizobial or mycorrhizal symbioses (Patten & Glick, 2002). In our findings, the root:shoot ratio was significantly magnified by mixed or single inoculations, while root biomass, length, branches, and root surface area in dual inoculations were significantly higher than in single inoculations (Table 1), demonstrating that mycorrhizal plants have more advanced root systems, as well as more potential for nutrient acquisition, and the co-occurrence of PGPR and mycorrhizae have benefits for plant growth and improve each other’s development. This shows that PGPR facilitate mycorrhizae hyphal growth when colonizing the host root (Artursson, Finlay & Jansson, 2006; Bianciotto et al., 2001; Hildebrandt et al., 2006; Jeffries et al., 2003; Pivato et al., 2009), increasing the amount and/or length of lateral roots (Chamam et al., 2013; Combes-Meynet et al., 2011) by mediating the hormone pathways and balances (Dodd et al., 2010; Moubayidin, Di Mambro & Sabatini, 2009; Peret et al., 2012; Stepanova & Alonso, 2009) and modifying root morphology (Aloni et al., 2006). It has been well documented that greater special root length is positively correlated with higher resource absorbing efficiency in root systems assimilating nutrients (particularly N and P) from the soil (Cantarel et al., 2015; Comas, Bouma & Eissenstat, 2002; Larson & Funk, 2016; Legay et al., 2014), which can also diminish nitrous oxide release and N extracted from the soil (Abalos et al., 2014; de Vries & Bardgett, 2016; Moreau et al., 2015).

### Soil property responses to inoculations

There is evidence of a tradeoff between different N forms (NO^3−^-N and NH^4+^-N) uptake among coexisting grass species that have no relationship with root morphology (Maire et al., 2009). Roots and their related mycorrhizal fungi regulate the long-term soil C pool by impacting organic substance decadence (Clemmensen et al., 2013; Phillips, Brzostek & Midgley, 2013) and promoting soil aggregation (Rillig et al., 2015). Additionally, the mixed inoculation of AMF and *Bacillus* significantly increased soil NH^4+^-N compared to the single inoculations of AMF or *Bacillus* in our results (Fig. 2B), which may be linked to PGPR’s potential in enhancing the NO^3−^-N assimilation rate (Sondergaard, Schulz & Palmgren, 2004) as well as the nitrification limitations of the AMF and PGPR combination (Arif et al., 2016). Consequently, the consumption and absorption of NH_4_^+^-N also was lower and better than NO_3_^−^-N (Hawkins, Johansen & George, 2000). Soil ALP and UE for both inoculations performed significantly better than the single symbiont in our study, and these results were consistent with recent research (El-Sawah et al., 2021; Zai et al., 2014). AMF may contribute to facilitating soil ALP activity, and its propagules have capacities to synthesize and release soil enzymes (Wang et al., 2006). Additionally, PGPR contribute to P mobilization (Krey et al., 2011), indicating that microorganisms increase the activity of phosphatase, as well as catalyze the hydrolysis of organic P into inorganic P that can be absorbed by plants. At the same time, they can secrete metabolites into the soil matrix during growth and reproduction, promote soil humification, accelerate the degradation of organic matter, and increase the content of organic matter (Mazzoncini et al., 2010). The increase of soil UE also promotes the N concentration in rhizosphere soil. Consequently, the promotion of soil enzyme activities could significantly facilitate the decomposition of organic matter and the remobilization of nutrients in rhizosphere soil (Zai et al., 2014). Inoculations significantly increased soil pH but decreased soil electrical conductivity compared with the control group, and there is similar evidence that Glomalin released by AMF can promote soil physicochemical properties (Mazzoncini et al., 2010).

## Conclusion

We evaluated the effects of AMF and PGPR on root morphology, plant growth, and soil properties. A dual inoculation of AMF and PGPR was the most effective for improving plant growth regulation, nutrient acquisition, and soil properties, and should be used as bio-fertilizer to promote local forage production and soil quality in the Qinghai-Tibet Plateau, and provide practical guidance for agricultural management.

## Supplemental Information

10.7717/peerj.13080/supp-1 Supplemental Information 1 Raw data.

## Additional Information and Declarations

### Data Availability

The following information was supplied regarding data availability: The raw data are available in the Supplemental Files.
